# 3-Chloro-*N*′-(2-hydroxybenzylidene) benzohydrazide: An LSD1-Selective Inhibitor and Iron-Chelating Agent for Anticancer Therapy

**DOI:** 10.3389/fphar.2018.01006

**Published:** 2018-09-07

**Authors:** Federica Sarno, Chiara Papulino, Gianluigi Franci, Jeanette H. Andersen, Bastien Cautain, Colombina Melardo, Lucia Altucci, Angela Nebbioso

**Affiliations:** ^1^Dipartimento di Medicina di Precisione, Università degli Studi della Campania Luigi Vanvitelli, Naples, Italy; ^2^Epi-C srl, Naples, Italy; ^3^Dipartimento di Medicina Sperimentale, Università degli Studi della Campania Luigi Vanvitelli, Naples, Italy; ^4^Marbio, The University of Tromsø – The Arctic University of Norway, Tromsø, Norway; ^5^Fundación MEDINA, Centro de Excelencia en Investigación de Medicamentos Innovadores en Andalucía, Granada, Spain

**Keywords:** novel therapies, cancer, epigenetics, iron chelating agent, chromatin remodeling

## Abstract

Despite the discovery and development of novel therapies, cancer is still a leading cause of death worldwide. In order to grow, tumor cells require large quantities of nutrients involved in metabolic processes, and an increase in iron levels is known to contribute to cancer proliferation. Iron plays an important role in the active site of a number of proteins involved in energy metabolism, DNA synthesis and repair, such as ribonucleotide reductase, which induce G0/S phase arrest and exert a marked antineoplastic effect, particularly in leukemia and neuroblastoma. Iron-depletion strategies using iron chelators have been shown to result in cell cycle arrest and apoptosis. Deferoxamine (DFO) was the first FDA-approved drug for the treatment of iron overload pathologies, and has also been recognized as having anticancer properties. The high cost, low permeability and short plasma half-life of DFO led to the development of other iron-chelating drugs. Pyridoxal isonicotinoyl hydrazone (PIH) and its analogs chelate cellular iron by tridentate binding, and inhibit DNA synthesis more robustly than DFO, demonstrating an effective antiproliferative activity. Here, we investigated the biological effects of a PIH derivative, 3-chloro-*N*′-(2-hydroxybenzylidene)benzohydrazide (CHBH), known to be a lysine-specific histone demethylase 1A inhibitor. We showed that CHBH is able to induce cell proliferation arrest in several human cancer cell lines, including lung, colon, pancreas and breast cancer, at micromolar levels. Our findings indicate that CHBH exerts a dual anticancer action by strongly impairing iron metabolism and modulating chromatin structure and function.

## Introduction

Despite continuous advances in screening, prevention and therapy, cancer remains a leading cause of death worldwide. Research efforts are therefore focused on finding new and specific therapeutic targets for personalized therapies that take into account individual variability (Collins and Varmus, 2015). However, precision medicine requires the use of precision drugs able to act only on the molecular determinant driving the specific disease, without producing adverse side effects. Thus, in recent years the drug discovery process has contributed to scientific advancements (Drews, 2000) by identifying novel small molecules with anticancer activity. High-throughput screening provides a new approach to drug discovery, allowing the screening of large libraries of heterogeneous compounds and the evaluation of their target modulation and biological effect. Cell death impairment is one of the six hallmarks of human cancer (Hanahan and Weinberg, 2000, 2011). A cancer cell retains the ability to undergo apoptosis, but signal transduction pathways are often silenced and therefore inactive. The remarkable potential of epigenetic drugs used in epigenetic therapy is their ability to reactivate these signals by correcting epigenetic defects. The last few years have seen growing scientific interest in the search for epi-targets to be exploited in diagnostics, prognostics and therapeutics. In addition, specific nutrients involved in different metabolic processes have been investigated as potential targets of anticancer drugs (Cheong et al., 2012; DeBerardinis and Chandel, 2016; Kalyanaraman, 2017). Iron, for example, is present inside cells in two main oxidation states, ferric (Fe^2+^) and ferrous (Fe^3+^), and is necessary for oxygen transport, energy transduction, macromolecule biosynthesis, and cell proliferation (Andrews, 2008; Pantopoulos et al., 2012). The rapid growth of tumor cells requires a large amount of iron, and the dysregulation of iron metabolism with increased uptake and decreased storage contributes to cancer progression. Recent studies show the exciting potential role of iron chelators in anticancer treatments (Richardson, 2002; Buss et al., 2003; Hatcher et al., 2009).

The siderophore deferoxamine (DFO) was the first molecule with a chelating mechanism used for thalassemia (Olivieri et al., 1998), and was later evaluated in clinical trials to assess its antiproliferative activity mediated by blocking G0/S phases for the treatment of neuroblastoma and leukemia (Lovejoy and Richardson, 2002; Richardson, 2005). However, DFO has two major drawbacks: oral inactivity due to hydrophilicity and a short half-life. Consequently, new compounds were developed as an alternative to DFO for cancer treatment, including deferiprone (DFP) (Jamuar and Lai, 2012; Martin-Bastida et al., 2017), triapine (Richardson, 2002; Myers et al., 2011) and hydrazones. Pyridoxal isonicotinoyl hydrazone (PIH) is a tridentate chelator which binds iron by carbonyl and phenolic oxygen, and imine nitrogen (Buss et al., 2002), with a ligand/iron ratio of 2:1. PIH was shown to act in cells by inhibiting DNA synthesis, as does DFO, but with greater efficiency (Richardson et al., 1995; Richardson and Ponka, 1998), principally as a result of its increased lipophilicity, which allows better penetration of the plasma membrane (Lovejoy and Richardson, 2003).

Here, by screening 23 commercial molecules we better characterized a PIH derivative, 3-chloro-*N*′-(2-hydroxybenzylidene)benzohydrazide (CHBH), known to be a lysine-specific histone demethylase 1A (LSD1) inhibitor (Sorna et al., 2013). LSD1 was the first enzyme found able to demethylate lysines 4 and 9 in histone H3 (Hayward and Cole, 2016; Maiques-Diaz and Somervaille, 2016) via a flavin-dependent monoamine oxidase mechanism (Forneris et al., 2005), thereby regulating gene expression (Miao and Natarajan, 2005; Cheng and Zhang, 2007). LSD1 is overexpressed in several human cancer cell lines (Kahl et al., 2006; Lim et al., 2010; Hayami et al., 2011; Niebel et al., 2014; Chen et al., 2017; Zou et al., 2017). We found that CHBH is able to induce cell proliferation arrest in a number of human cancer cell lines, including lung, colon, pancreas and breast cancer, while sparing normal cells.

Our findings show that CHBH is able to induce selective cancer cell death through both chromatin modulation and elimination of intracellular iron pools, making it a promising anticancer agent.

## Materials and Methods

### Cell Lines

Cell lines were tested and authenticated following the manufacturer’s instructions: DSMZ for NB4 and ATCC for all the others. HCT-116 and HT-29 (colon cancer), MCF7 (breast cancer), A549 (lung cancer), MiaPaCa (pancreas carcinoma), and A2058 (melanoma) cells were propagated in Dulbecco’s modified Eagle’s medium (Euroclone, Milan, Italy) with 10% fetal bovine serum (FBS) (Euroclone), 2 mM L-glutamine (Euroclone) and antibiotics (100 U/ml penicillin, 100 mg/ml streptomycin; Euroclone). NB4 (acute promyelocytic leukemia) and K562 (chronic myelogenous leukemia) cells were propagated in RPMI-1640 medium containing 4.5 g/L glucose (Euroclone) supplemented with 10% FBS (Euroclone), 100 U/mL penicillin–streptomycin (Euroclone) and 2 mM L-glutamine (Euroclone). MRC5 (normal human lung) cells were propagated in Eagle’s minimum essential medium (Euroclone) supplemented with 10% FBS (Euroclone), and 10 μg/ml gentamicin solution (Euroclone).

### Cell Cycle and Cell Death Analysis

HCT-116 and NB4 cells were treated for 24 and 72 h with CHBH (25 μM), or with SAHA (5 μM) or PKF118-310 (1 μM), used as positive controls of cell death. After treatment, cells were collected, then centrifuged (1,200 rpm for 5 min) and suspended in a solution containing 1X PBS, 0.1% sodium citrate, 0.1% NP40, and 50 mg/mL propidium iodide. After 20 min of incubation at room temperature in the dark, cell cycle and cell death were evaluated by FACS (FACSCalibur, BD Biosciences, San Jose, CA, United States) and analyzed by FACS with Cell Quest Pro software (BD Biosciences). Cell death was measured as a percentage of cells in pre-G1 phase.

### MTT Assay

The viability of cells was determined using the standard MTT [3-(4,5-dimethylthiazol-2-yl)-2,5-diphenyltetrazolium bromide] assay. Briefly, 5 × 10^4^ cells/well were plated in a 24-well plate and treated, in triplicate, with CHBH at different concentrations for 24 and 72 h. MTT solution was added for 3 h at 0.5 mg/mL, the purple formazan crystals were dissolved in DMSO (Sigma-Aldrich, St. Louis, MO, United States) and the absorbance was read at a wavelength of 570 nm with a TECAN M-200 reader (Tecan, Männedorf, Switzerland).

### Protein Histone Extraction

HCT-116 cells were treated with 25 μM CHBH for 24 and 48 h. After treatment, cells were harvested and washed twice with PBS (Euroclone). Cells were then lysed in triton extraction buffer (TEB) containing PBS with 0.5% Triton X-100 (v/v), 2 mM phenylmethylsulfonyl fluoride (PMSF), 0.02% (w/v) NaN3 at a cell density of 10^7^ cells/mL for 10 min on ice and centrifuged (2,000 rpm at 4°C for 10 min). The supernatant was removed and the pellet washed in half the volume of TEB and centrifuged another time. The pellet was suspended in 0.2N HCl overnight at 4°C on a rolling table. The samples were centrifuged at 2,000 rpm for 10 min at 4°C and the supernatant recovered. The histone protein was determined using a Bradford assay (Bio-Rad, Milan, Italy).

### Western Blotting

Western blotting analysis was performed following the recommendations of antibody suppliers and loading 8 μg of histone extracts on 15% polyacrylamide gels. Antibodies used were: H3K9me2, H3K27me3, H3K4me3, and H3K9/14ac (Diagenode, Liège, Belgium); histone H4 (Abcam, Cambridge, United Kingdom). Semi-quantitative analysis was performed using ImageJ software.

### Enzymatic Assay

CHBH activity on KDM4A enzyme was evaluated by KDM4A Inhibitor Enzymatic Assay Kit (Epi-C srl, Naples, Italy). CHBH was incubated for 30 min at 37°C with 13.5 μL buffer, 6 μL substrate and 4.5 μL human recombinant protein in a 96-well black half-area plate. Next, 24 μL developer 1 solution and 6 μL developer 2 solution were added in each well. After 30 min at room temperature the fluorescence was read with a TECAN M-200 reader at excitation wavelength of 370 nm and emission wavelength of 470 nm. The experiment was performed in triplicate.

### EnSpire Binding Assay

KDM4A-GST enzyme was purified by *Escherichia coli* BL21 bacteria after transfection with PGEX-4T-1-KDM4A plasmid. One bacterial colony was grown in LB Broth medium (Sigma-Aldrich) supplemented with antibiotics (100 μg/mL ampicillin) in a shaking incubator overnight. When optical density was in a range between 0.6 and 0.8, protein expression was induced by isopropyl-β-D-1-thiogalactopyranoside (AppliChem, Milan, Italy) at 200 μM concentration for 7 h. The bacteria were lysed by sonication (Bioruptor, Diagenode) in lysis buffer containing PBS with 1 mM dithiothreitol (DTT) (AppliChem), 0.5 mM PMSF (AppliChem) and mini protease inhibitor cocktail (PIC) 1x (Roche, Monza, Italy). The enzyme was purified using a GSTrap 4B column (GE Healthcare Life Sciences, Milan, Italy). The purified recombinant KDM4A (40 mg/mL) was then dialysed in water solution (100 mM NaCl, 1 mM DTT, 50 mM sodium acetate pH 6.0). For the binding assay, the enzyme was diluted in sodium acetate solution (20 mM pH 6.0) to obtain a final concentration of 150 μg/mL, and 15 μL of this solution was put in 384 high-performance optical microplate wells. The plate was centrifuged at 800 rpm for 1 min and incubated overnight at 4°C. The following day, the immobilized enzyme was washed four times using 25 μL PBS, centrifuging the plate at 800 rpm for 1 min after each wash. After the last wash the plate was incubated at room temperature for 3 h, and during the last 30 min was placed in the EnSpire instrument (PerkinElmer, Milan, Italy) to equilibrate before the binding assay. Next, the baseline was read and 15 μL of either CHBH or positive or negative control was added into the plate at 100, 50, and 25 μM (in PBS solution with 0.1% DMSO). Finally, the plate was reloaded into the EnSpire instrument to start final reads.

### Calcein-AM Assay

Chronic myelogenous leukemia K562 cells were loaded at a density of 1 × 10^6^/mL in normal medium and treated with calcein-acetoxymethyl ester (calcein-AM) (Life Technologies, Carlsbad, CA, United States) at 0.125 μM for 15 min at 37°C and 5% CO_2_. Cells were then washed three times with PBS and three times with distilled water. The pellet was resuspended in 500 μL water, lysed by thermic shock and centrifuged for 30 min at 1,300 rpm. The supernatant was recovered and the calcein read using a TECAN M-200 reader in a 96-well black half-area plate at excitation wavelength of 488 nm and emission wavelength of 516 nm. Ammonium iron(III) sulfate dodecahydrate (AIS) (Fluka, Bucharest, Romania) was added at 30 and 100 μM final concentration to quench the calcein. CHBH and positive control (EDTA) at 100 μM were co-administered with AIS to evaluate iron-chelating activity (Cabantchik et al., 1996; Epsztejn et al., 1997; Konijn et al., 1999).

### Statistical Analysis

Data were presented as the mean ± SD of biological triplicates. Differences between the treatment groups and controls were compared using one-way analysis of variance (ANOVA) and Dunnett’s multiple-comparison test. Differences between groups were considered to be significant at a *p-* value of < 0.05. Statistical analyses were performed using GraphPad Prism 6.0 software (GraphPad Software, Inc., San Diego, CA, United States).

## Results

### CHBH Induces Cell Death in Human Cancer Cell Lines

**FIGURE 1:**
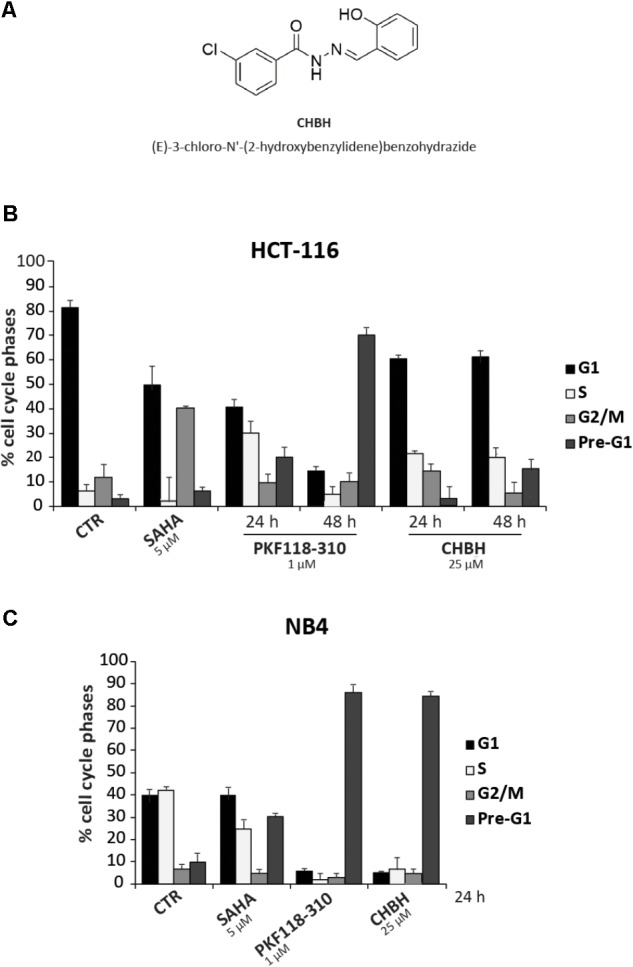
Impact of CHBH on cell cycle of HCT-116 and NB4 cells. **(A)** Chemical structure of CHBH; **(B,C)** FACS analysis showing that CHBH impairs cell cycle progression and induces cell death in HCT-116 and NB4 cells. The total amount of cells in G1, S, G2/M, and pre-G1 is 100%. Values are mean ± SD of biological triplicates.

We screened a panel of 23 synthetic compounds (**Supplementary Figure S1**) for their cytotoxic activity in two human cancer cell lines, and identified CHBH as a promising anticancer drug candidate (**Figure 1A**). Colon cancer HCT-116 and acute promyelocytic leukemia NB4 cells were left untreated or treated at the indicated times with 25 μM CHBH (**Figures 1B,C**) and with two well-characterized anticancer drugs, SAHA and PKF118-310 (Nebbioso et al., 2005; Franci et al., 2017). After incubation, cell cycle progression was determined by FACS analysis. Our data revealed that CHBH affected HCT-116 distribution over cell cycle phases within 48 h, reducing G1 phase and increasing S phase (**Figure 1B**). Furthermore, CHBH-induced cell death, expressed as a percentage of cells in pre-G1 phase, increased in a time-dependent manner. Compared to untreated cells, CHBH induced increased cell death at 48 h, although not to the same level as PKF118-310 (≈20 vs. ≈70%, respectively).

In the NB4 cell line, CHBH had a marked impact on cell cycle progression after 24 h, inducing a robust (≈80%) cell death similar to or higher than that mediated by PKF118-310 and SAHA, respectively (**Figure 1C**). Taken together, these initial findings strongly suggest a promising CHBH-mediated antiproliferative effect in these two solid and hematological cancer cell systems.

**FIGURE 2:**
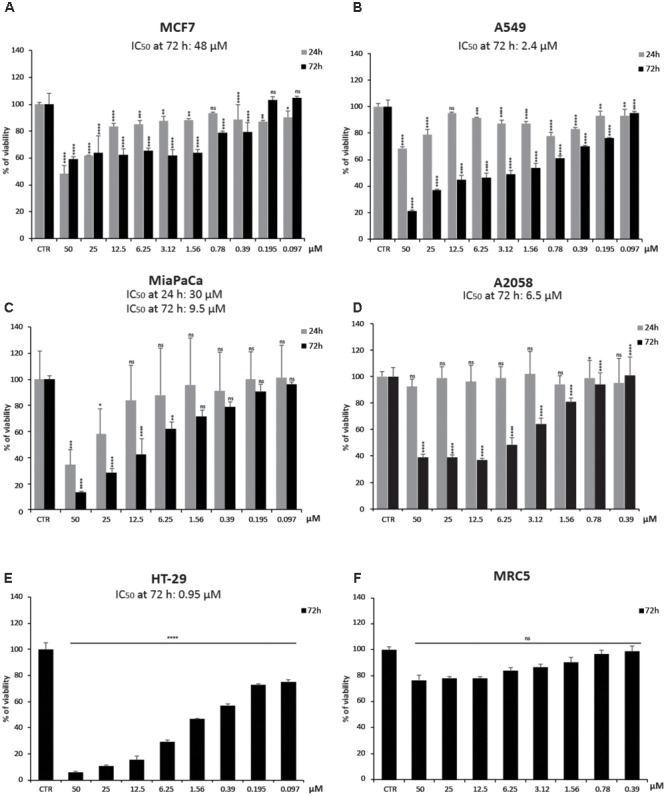
Impact of CHBH on cell proliferation. **(A–E)** Cell growth rates determined by MTT after treatment with indicated concentrations of CHBH for 24 h (gray bars) and 72 h (black bars) show its anticancer activity. IC50 values show different cellular sensitivity to CHBH; **(F)** CHBH did not affect cell viability of normal MRC5 cells. Absorbance was read at a wavelength of 570 nm. Values are mean ± SD of biological triplicates. ^∗∗∗∗^*p*-value ≤ 0.0001, ^∗∗∗^*p*-value ≤ 0.001, ^∗∗^*p*-value ≤ 0.01, ^∗^*p*-value ≤ 0.05, ns *p*-value > 0.05 vs. control cells.

To better investigate CHBH cytotoxicity, we tested the viability of other human cancer cell lines after exposure to the compound (**Figure 2**). By MTT colorimetric assay, we evaluated the effect of different doses of CHBH in MCF7 (breast), A549 (lung), MiaPaCa (pancreas), A2058 (melanoma), and HT-29 (colon) cancer cell lines after 24 and/or 72 h treatment. The viability of all cell lines was affected by CHBH incubation in a time- and dose-dependent manner. As shown by IC_50_ values at 72 h, colon cancer HT-29 cells were the most sensitive (IC_50_ ≈0.95 μM; **Figure 2E**), followed in decreasing sensitivity by A549 (IC_50_ ≈2.4 μM; **Figure 2B**), A2058 (IC_50_ ≈6.5 μM; **Figure 2D**), and MiaPaCa (IC_50_ ≈9.5 μM; **Figure 2C**) cell lines. In breast cancer MCF7 cells, CHBH displayed moderate cytotoxicity with IC_50_ ≈48 μM (**Figure 2A**). Interestingly, no significant effect on viability was observed in the MRC5 normal lung cell line. Even when MRC5 cells were exposed to the highest dose of CHBH, no significant difference was observed, compared to the control (**Figure 2F**). Normal cell viability was not affected as it was in the other cell lines, suggesting that the CHBH-induced antiproliferative effect may be a cell type-independent cancer response, occurring both in hematological and solid cancer cell lines but not in normal cells.

### CHBH Impairs Histone Methylation and Acetylation Levels

**FIGURE 3:**
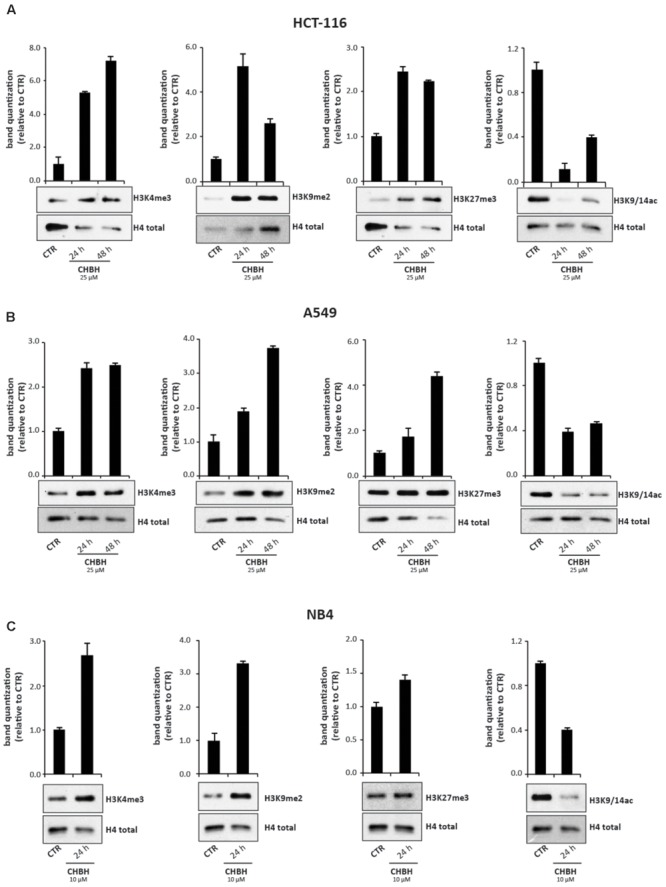
Impact of CHBH on methylation and acetylation levels of histone H3. Western blot analysis of expression levels of H3K4me3, H3K9me2, H3K27me3, and H3K9/14ac in **(A)** HCT-116 **(B)** A549 and **(C)** NB4 cells after treatment with CHBH at indicated times and concentrations. H4 antibody was used for protein normalization. Values are mean ± SD.

We next performed Western blotting analysis to confirm that CHBH acts as an inhibitor of LSD1 enzyme. HCT-116 cells were treated with 25 μM CHBH for 24 and 48 h, and levels of H3K4me3, H3K9me2, and H3K27me3 were determined on histone extracts (**Figure 3A**). LSD1 inhibition resulted in a general gain of histone H3 methylation. Specifically, CHBH treatment induced in a time-dependent manner an increase in H3K4me3 peaking at over sevenfold at 48 h and an increase in H3K9me2 at over fivefold at 24 h. Furthermore, H3K27me3 signal increased by and remained stable at twofold within 48 h. Consistent with the above findings, CHBH treatment reduced global acetylation levels of histone H3 (**Figure 3A**, right panel), highlighting CHBH-induced chromatin modulation. We obtained similar findings in other solid (A549; **Figure 3B**) and hematological (NB4; **Figure 3C**) cancer cell lines. Given the strong impact of the compound on cell cycle progression (**Figure 1C**), NB4 cells were treated with 10 μM CHBH for 24 h. Taken together, these data indicate that, via LSD1 inhibition, CHBH triggers wide-ranging histone alterations responsible for the activation or repression of specific loci. Further investigations will be required to understand which genes are involved in the response to CHBH.

### KDM4A Is Not a Target of CHBH

**FIGURE 4:**
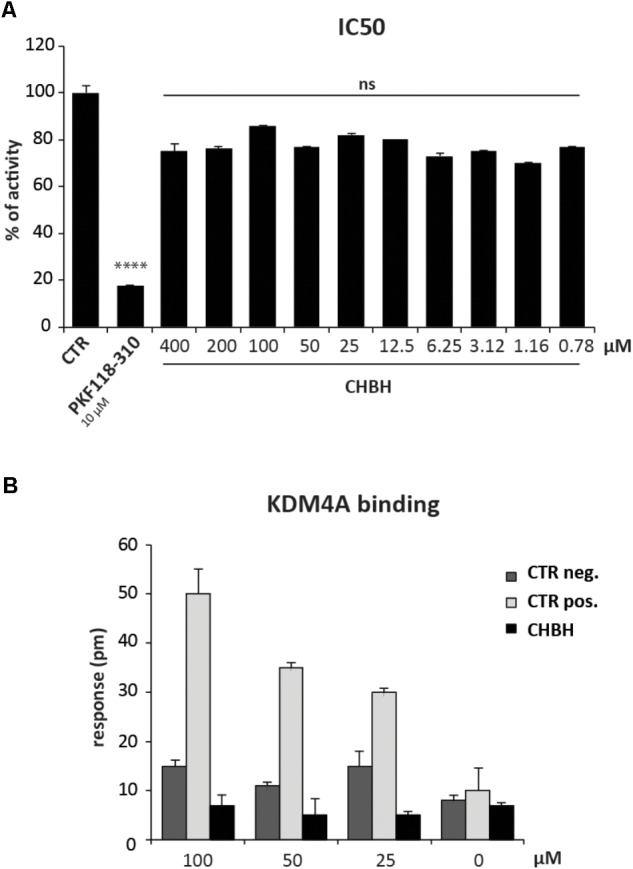
CHBH does not target KDM4A. **(A)** KDM4 activity evaluated by *in vitro* enzymatic assay in presence of 10 different concentrations of CHBH. PKF118-310 at 10 μM was used as positive control. **(B)** Binding between KDM4A and CHBH evaluated by EnSpire label-free biochemical assay. The response was measured in picometers (pm) as the difference in wavelength. Values are mean ± SD of biological triplicates. ^∗∗∗∗^*p*-value ≤ 0.0001, ns *p*-value > 0.05 vs. control.

To better characterize the enzymatic selectivity of CHBH, we investigated its ability to inhibit another lysine demethylase, KDM4A, by using an *in vitro* enzymatic assay (**Figure 4A**) and a binding assay (**Figure 4B**). We performed the *in vitro* enzymatic assay for KDM4A as described in Franci et al. (2017). Briefly, KDM4A-mediated H3K9me3 demethylation leads to formaldehyde production. The reaction between formaldehyde, ammonia, and acetoacetanilide produces a fluorescent adduct which is detected at excitation wavelength of 370 nm and emission wavelength of 470 nm. If the compound under investigation acts as an inhibitor, the reaction is blocked and the fluorescence signal decreases. As reported in **Figure 4A**, we tested KDM4A activity against 10 different concentrations of CHBH, ranging from 400 to 0.78 μM. Unlike PKF118-310, our positive control of inhibition, CHBH treatment did not result in significant modulation of KDM4A activity.

We confirmed these results by evaluating the binding between CHBH and KDM4A by EnSpire label-free biochemical assay (**Figure 4B**). This technique allows the detection of interactions in real time by measuring the change in wavelength of refracted light in response to binding between KDM4A, previously immobilized onto a plate, and the molecules being tested. The difference in wavelength (measured in picometers) is reported as the response. As shown in **Figure 4B**, incubation of CHBH in wells containing immobilized KDM4A resulted in minimal response compared to the changes in wavelength observed in presence of the positive control at the same concentration. CHBH response was similar to the negative control. These data confirm that CHBH does not act on KDM4A enzyme and indicate that the compound is a selective inhibitor of LSD1.

### CHBH Dequenches Calcein–Iron Complex

**FIGURE 5:**
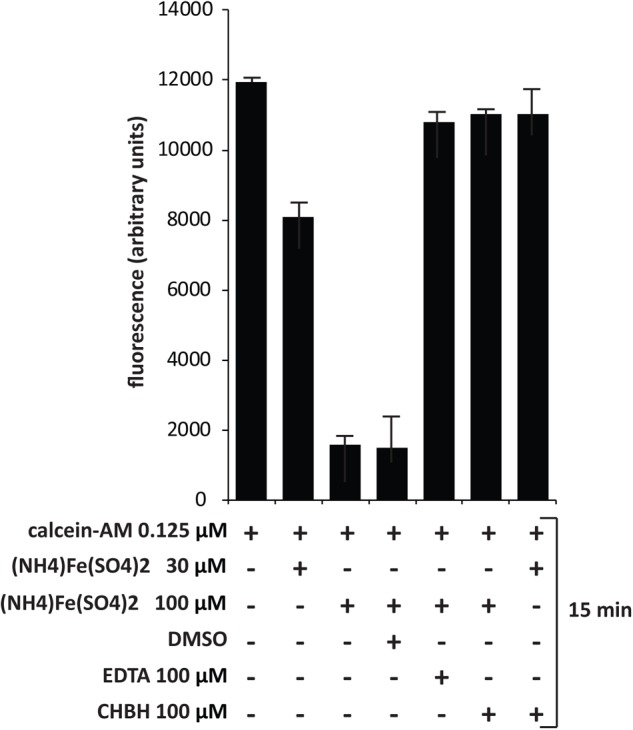
Dequenching of calcein–iron complex by CHBH. Fluorescence assay of K562 cells treated with calcein-AM, (NH_4_)_2_Fe(SO_4_)_2_, DMSO (negative control), EDTA (positive control) and CHBH at the indicated time. The iron-chelating property of CHBH is comparable to that of EDTA. Values are mean ± SD of biological triplicates.

Since CHBH is a derivative of PIH, we investigated its activity as an iron chelator in order to better assess its anticancer cytotoxicity. We evaluated the ability of CHBH to scavenge iron by measuring intracellular fluorescence with the iron-sensitive probe calcein (**Figure 5**). Chronic myelogenous leukemia K562 cells were treated according to the basic concepts of calcein fluorescence assay (Cabantchik et al., 1996) to determine the iron-chelating ability of CHBH to sequester iron from cells. Specifically, K562 cells were treated with the cell-permanent dye calcein-AM, which is converted inside the cell to a green-fluorescent calcein, detected at excitation wavelength of 488 nm and emission wavelength of 516 nm. We observed that calcein fluorescence was quenched by iron addition in a dose-dependent manner. CHBH fully restored the quenching of calcein fluorescence by iron to a similar degree to that of the positive control, EDTA. This finding shows that CHBH is a strong iron-chelating agent, accounting - at least in part - for its antiproliferative action in the cancer cell lines investigated in this study.

## Discussion

The six hallmarks of cancer defined by Hanahan and Weinberg as capabilities acquired during malignant transformation that allow cancer cells to survive, proliferate, and disseminate (Hanahan and Weinberg, 2000, 2011) are the main targets of current anticancer therapies. The definition of cancer hallmarks has made a significant contribution to the rapid growth of drug discovery research into new molecules able principally to induce cancer cell death. In recent years, small molecule drug discovery in the field of oncology has focused on identifying compounds with well-characterized mechanisms of action (Fouad and Aanei, 2017; Hyman et al., 2017). However, the increased awareness that effective cancer treatment is highly dependent on drug combinations has driven the development of drugs that are simultaneously able to affect different biological processes in order to optimize clinical benefits for patients (Hoelder et al., 2012). The recognition that cancer hallmarks are orchestrated by aberrant epigenetic alterations and the fact that many of these alterations are druggable has propelled the discovery of small molecules targeting different classes of epigenetic enzymes (Falahi et al., 2014; Schnekenburger et al., 2016). Therapeutic strategies using epigenetic drugs, which control several biological processes, provide a multi-target approach against the hallmarks of cancer (Kelly et al., 2010; Nebbioso et al., 2011; Block et al., 2015).

We screened a number of commercially available molecules for their potential cytotoxic activity in solid and hematological cancer cell lines, and identified CHBH as a compound able to affect chromatin structure as well as iron metabolism. CHBH reduced the viability of a panel of cancer cell lines with IC_50_ values in the micromolar range, suggesting its beneficial application in multiple settings. Interestingly, we found that CHBH did not affect the viability of a normal cell line. Thus, we provide promising initial evidence that CHBH is cytotoxic for cancer cells but not for normal cells. CHBH is known to be an inhibitor of LSD1. LSD1 is a demethylase that converts H3K4me2 or H3K9me2 into mono- or unmethylated forms, inhibiting the expression of its target genes (Shi et al., 2004; Amente et al., 2013; Hyun et al., 2017). When inhibited, LSD1 increases H3K4 methylation levels leading to the expression of tumor suppressor genes (Rotili and Mai, 2011; Jin et al., 2013). LSD1 is overexpressed in several human cancer cells, indicating its key role in tumor cell growth and survival (Hayami et al., 2011; Liu et al., 2018). We therefore speculated that CHBH may exert its anticancer action by inhibiting LSD1 activity. Consistent with this hypothesis, our findings showed that CHBH treatment results in a general increase in histone H3 methylation levels followed by a reduction in H3 acetylation levels. The effects of CHBH on methylation and acetylation levels of histone H3 correlated with its impact on HCT-116 viability at 24 and 48 h. Further research is necessary to determine which genes are regulated upon CHBH-mediated chromatin remodeling in order to identify oncogenes and tumor suppressor genes responsible for CHBH-induced cell death. In addition, data from our KDM4A studies showed that CHBH may be an LSD1-selective inhibitor. This property would make it a suitable candidate for future studies investigating the role of LSD1 in both biological processes and LSD1-driven diseases.

Cell death can also be due to altered iron metabolism (Torti and Torti, 2013). Iron levels increase in cancer cells, thereby sustaining their growth and survival (Manz et al., 2016). By fluorescence assay, we demonstrated that CHBH, a derivative of PIH, is an iron-chelating agent with comparable efficacy to EDTA, thus pointing to its dual anticancer action. Our findings provide initial evidence that the cancer-selective cytotoxic effect of CHBH is due partly to the rearrangement of chromatin by LSD1 inhibition, which may lead to reactivation of tumor suppressor genes, and partly the removal of iron from cancer cells as a result of its iron-chelating property. Both actions require further clarification to gain a greater insight into the molecular mechanisms underlying the antioncogenic effects of CHBH. In conclusion, we show for the first time that this compound strongly reduces cancer cell proliferation both by impairing iron metabolism and modulating chromatin structure via LSD1-selective inhibition. One of the emerging challenges in pharmacology is indeed the design of small molecules able to inhibit simultaneously several molecular targets in malignant transformation, where a plethora of altered pathways determine the hallmarks of cancer (Alvarez et al., 2011; Nebbioso et al., 2011; Ganesan, 2016). The use of a single drug eliciting two different effects may represent a promising approach for cancer therapy and overcome the drawbacks of combination therapies in terms of drug resistance, toxicity, and side effects.

## Author Contributions

AN and LA: conception of the work. FS, CP, GF, JA, BC, and CM: experimental study, data analysis, and interpretation. AN and FS: writing of the manuscript. All authors gave final approval of the manuscript.

## Conflict of Interest Statement

The authors declare that the research was conducted in the absence of any commercial or financial relationships that could be construed as a potential conflict of interest.

## Supplementary Material

FIGURE S1 Cytotoxic activity of a panel of 23 synthetic compounds. FACS analysis of 23 compounds screened in **(A,B)** HCT-116 and **(C)** NB4 cell lines. SAHA and PKF118-310 were used as controls. The total amount of cells in G1, S, G2/M, and pre-G1 is 100%. Values are mean ± SD of biological triplicates.
