# Supplementary figures and images for: 3-Chloro-N′-(2-hydroxybenzylidene) benzohydrazide: An LSD1-Selective Inhibitor and Iron-Chelating Agent for Anticancer Therapy
Source: Front Pharmacol. 2018 Sep 7;9:1006. doi: 10.3389/fphar.2018.01006 (PMC6137965; doi:10.3389/fphar.2018.01006)

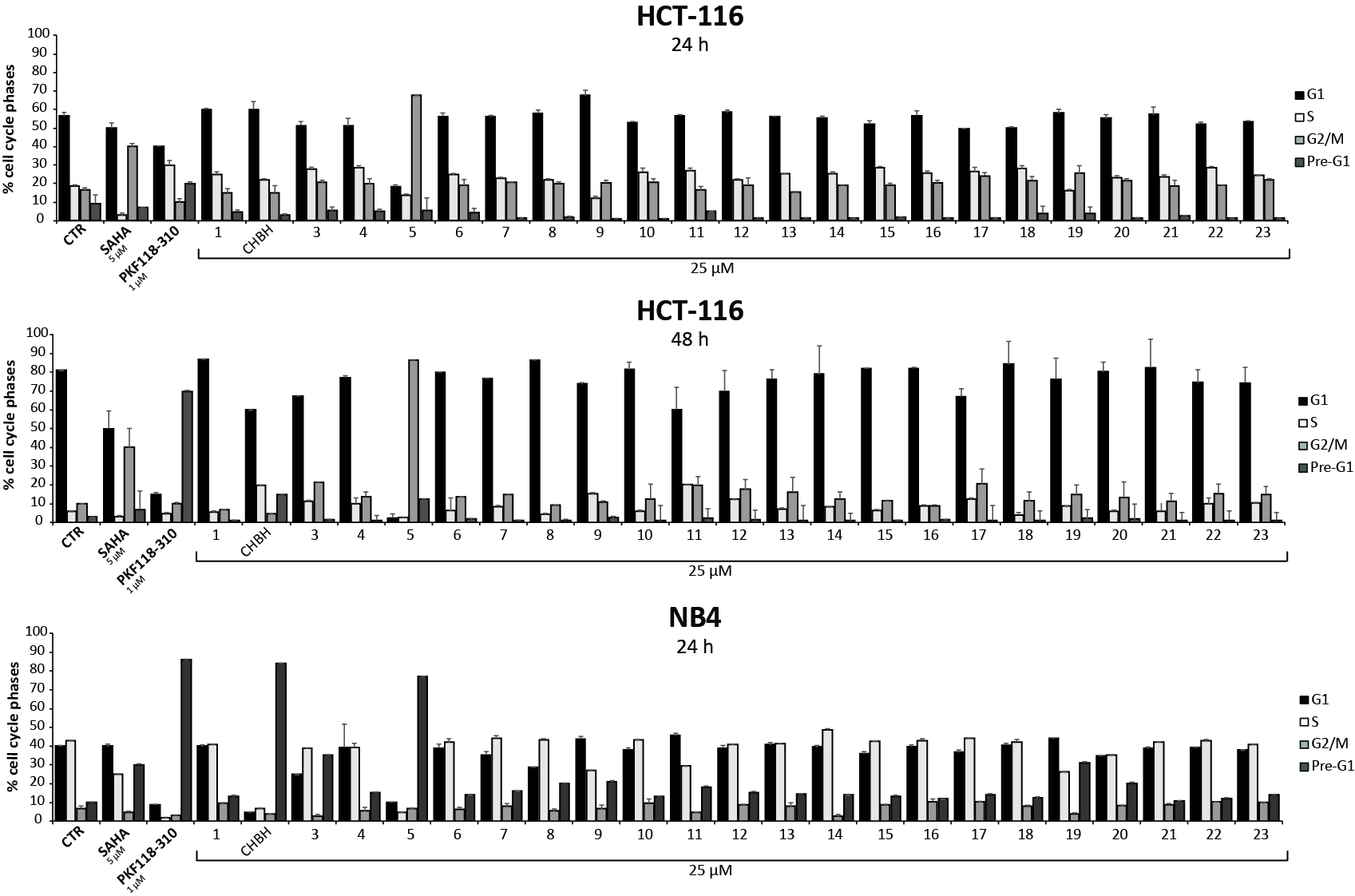

Supplement: FIGURE S1 — Cytotoxic activity of a panel of 23 synthetic compounds. FACS analysis of 23 compounds screened in (A,B) HCT-116 and (C) NB4 cell lines. SAHA and PKF118-310 were used as controls. The total amount of cells in G1, S, G2/M, and pre-G1 is 100%. Values are mean ± SD of biological triplicates. [file Image_1.TIF]
